# Maternal body mass index and risk of fetal overgrowth in women with gestational diabetes Mellitus in Southeast China: a retrospective cohort study

**DOI:** 10.1186/s13098-023-01093-y

**Published:** 2023-06-08

**Authors:** Lihua Lin, Jianhang Wu, Libo Xu, Jianqi Fang, Juan Lin

**Affiliations:** 1 Department of Healthcare, Fujian Maternity and Child Health Hospital, Affiliated Hospital of Fujian Medical University, Fuzhou, 350001 Fujian Province P.R. China; 2 Department of Ultrasonography, Fujian Maternity and Child Health Hospital, Affiliated Hospital of Fujian Medical University, Fuzhou, 350001 Fujian Province P.R. China; 3 Department of Computer center, Fujian Maternity and Child Health Hospital, Affiliated Hospital of Fujian Medical University, Fuzhou, 350001 Fujian Province P.R. China; 4 Department of women’s health care, Fujian Maternity and Child Health Hospital, Affiliated Hospital of Fujian Medical University, Fuzhou, 350001 Fujian Province P.R. China

**Keywords:** Body mass index change, Large for gestational age, Gestational diabetes mellitus

## Abstract

**Background:**

To investigate the relationship between body mass index (BMI) changes and large for gestational age (LGA) in women with gestational diabetes mellitus (GDM).

**Methods:**

A retrospective cohort study including 10,486 women with GDM was conducted. A dose‒response analysis of BMI changes and the occurrence of LGA was performed. Binary logistic regressions were performed to assess crude and adjusted odds ratios (ORs) and 95% confidence intervals (CIs). Receiver operating characteristic (ROC) curves and areas under the curve (AUCs) were used to assess the ability of BMI changes to predict LGA.

**Results:**

The probability of LGA increased with increasing BMI. The risk of LGA increased across the BMI change quartiles. The BMI change remained positively associated with the risk of LGAafter stratification analysis. The AUC was 0.570 (95% CI: 0.557 ~ 0.584)in the entire study population, and the best optimal predictive cut-off value was 4.922, with a sensitivity of 0.622 and a specificity of 0.486. The best optimal predictive cut-off value decreased from the underweight group to the overweight and obese group.

**Conclusions:**

BMI changes are related to the risk of LGA and may be a useful predictor of the incidence of LGA in singleton pregnant women with GDM.

**Supplementary Information:**

The online version contains supplementary material available at 10.1186/s13098-023-01093-y.

## Background

The reported prevalence of gestational diabetes mellitus (GDM) is rapidly increasing worldwide [1]. The total incidence of GDM is approximately 15% in mainland China [2]. It is well established that GDM poses a great threat to mothers and their offspring and has not only short-term but also long-term effects, including large for gestational age (LGA). LGA describes excessive foetal growth, defined as newborn birth weights at or above the 90th percentile for gestational age. For mothers, LGA can lead to prolonged labour and an increased risk of caesarean section, obstructed shoulder delivery, postpartum haemorrhage, and birth trauma, and LGA infantsare more prone to foetal hypoxia and intrauterine death and are at higher risk of developing diabetes, obesity, and metabolic syndrome in adulthood [3]. Maternal prepregnancy overweight or obesity, excessive gestational weight gain, and GDM have been defined as independent risk factors for LGA [4–7]. A complex interplay exists among maternal prepregnancy overweight or obesity, excessive gestational weight gain, and GDM on adverse pregnancy outcomes, including foetal overgrowth [8]. Therefore, it is difficult to tease apart the impacts of prepregnancy overweight or obesity, excessive gestational weight gain, and GDM on birth weight [9]. In particular, excessive gestational weight gain can complicate pregnancies and even promote GDM [10]. Previous studies assessing only women with GDM reported an increased risk of LGA with excessive gestational weight gain [11–13]. The Institute of Medicine(IOM) and Chinese guidelines for gestational weight gain do not distinguish women with GDM [14, 15]. Whether these guidelines are suitable for Chinese pregnant women withGDM is still unclear, and there is no agreement on the optimal gestational weight gain for women with GDM. These guidelines recommend weight gain ranges during pregnancy on the basis of the prepregnancy body mass index (BMI) categories. However, extremely tall and extremely short women can have identical BMIs, reflecting completely different body sizes. Therefore, the use of gestational weight gain alone may introduce a bias, as it may not reflect body size changes during pregnancy.

BMI changes, taking weight gain changes and body height into account, can be a better measure reflecting pregnancy-related weight gain as well as foetal growth. BMI changes have been reported in the obstetrics maternal gestational weight gain management field, including in overweight women [16] and women with GDM [17], preventing spontaneous preterm birth [18] and predicting macrosomia [19–21]. However, no study has focused on the optimal BMI change for preventing LGA among women with GDM.

We hypothesized that the BMI change during pregnancy may be a better predictor for foetal overgrowth assessment, which reflects the gestational weight gain measure. Thus, this study aimed to investigate whether the BMI change during pregnancy was related to LGA.

## Materials and methods

### Study population

The study data were retrieved from the Fujian Maternity and Child Health Hospital medical system, and women who were diagnosed with GDM and delivered a single neonate at a gestational age of ≥ 28 weeks between January 2015 and December 2019 were included. The eligibility criteria included women who received perinatal care during the whole pregnancy and underwent a 75-g oral glucose tolerance test(OGTT) between 24 and 28 weeks of gestation. We excluded women aged less than 18 years, those with prepregnancy diabetes, prepregnancy hypertension, chronic heart disease, kidney disease, and autoimmune disease, those who experienced stillbirth or miscarriage, those whose infants had birth defects, and those with twin or multiple births. We also excluded the following patientsfrom our analysis: those who lacked information on maternal weight, gestational age, parity, gravidity, birth weight, andOGTT values and those aged younger than 18 years or older than 45 years. This study was approved by the Ethics Committee of Fujian Maternity and Child Health Hospital [2020(NO.2049)]. Informed consent was not required since the current study was conducted through a retrospective review of medical records.

### Data collection

All the data regarding demographic and obstetric characteristics were collected from the clinical medical records. Demographic information and detailed clinical data were extracted from prenatal health visit records, and pregnancy outcomes were collected for review fromthe postpartum chart. The main pregnancy outcome in the current study was LGA.

### Definitions

We used self-reported prepregnancy weight and measured height at the first-trimester prenatal visit to calculate prepregnancy BMI(kg/m^2^), which was classified as underweight(BMI < 18.5 kg/m^2^), normal weight(18.5 kg/m^2^ ≤ BMI < 24 kg/m^2^), overweight (24.0 kg/m^2^ ≤ BM < 28 kg/m^2^) and obese (BMI ≥ 28 kg/m^2^) based on the Chinese adult weight standard [22]. Due to the small number of obese women, we merged them into the overweight group and named it the overweight and obesity group. Gestational weight gain was calculated as maternal weight at delivery minus the prepregnancy weight and was classifiedas below, above, and within the recommendations according to weight monitoring and evaluation of Chinese women during pregnancy [14]. The BMI change was expressed as the BMI at delivery minus the prepregnancy BMI. GDM was diagnosed by a 75-g oral glucose tolerance test at 24 to 28 gestational weeks when one or more of the parameters exceeded the following criteria: a fasting plasma glucose level of 5.1 mmol/L, a 1-h plasma glucose level of 10.0 mmol/L, and a 2-h plasma glucose level of 8.5 mmol/L [23]. LGA was defined as a birth weight above the 90th percentile based on sex and gestational age [8]. We selected maternal glycaemic control and serum triglyceride levels during the third trimester as covariates, as the two have significant impacts on foetal weight [24–26].Other covariates were also assessed: maternal age, employment, educational level, gravidity, and parity.

### Statistical analysis

Continuous variables with a normal distribution are shown as the mean ± standard deviation or medians(interquartile ranges), and categorical variables are shown as the frequency(percentage). Clinical characteristic analysis according to BMI change quartiles were compared using analysis of variance(ANOVA) for continuous variables with a normal distribution, the Kruskal‒Wallis for continuous variables with askewed distribution, and chi-square tests for categorical variables. The dose‒response analysis of BMI changes and the occurrence of LGA was explored using the restricted cubic spline model. Binary logistic regressions were performed to assess crude and adjusted odds ratios (ORs) and 95% confidence intervals (CIs) of the associations of BMI changes with LGA (quartiles, per unit, and per standard deviation (SD)) and were modified by three models. We tested the linear trends of increasing BMI change quartiles by assigning their median. Subgroup analyses to evaluate the ORs of LGA stratified by advanced maternal age (yes, no), educational level(below university, university and above), employment(employed, unemployed), gravidity(1,2, ≥ 3), parity(primiparous, multiparous, prepregnancy BMI(underweight, normal weight, overweight and obesity), infant sex(girl, boy). Interaction across subgroups was tested using the likelihood ratio test. Receiver operating characteristic (ROC) curves and areas under the curve (AUCs) were used to assess the ability of BMI changes to predict LGA.

All analyses were performed with the statistical software package R software, version 4.4.2. The level of statistical significance was defined as P < 0.05.

## Results

### Basic characteristics of the study population

A total of 16,803 pregnant women with GDM received prenatal visits and delivered a single neonate at Fujian Maternity and Child Hospital from January 2015 to December 2019. We first excluded the following women: women aged less than 18 or more than 45 years(n = 36), those who experienced miscarriage, stillbirth and neonatal death(n = 156), those whose infants had birth defects (n = 423), those with prepregnancy hypertension or diabetes (n = 31), chronic heart, kidney disease, or autoimmune disease(n = 429), those with twin or multiple births(n = 255), those with postterm pregnancy(n = 11), those with missing data for prepregnancy weight (n = 98) or without at least onegestational weight measurement during the first, second, and third trimesters and within one week of delivery (n = 4237), and those lacking maternal weight, gestational age, parity, gravidity, birth weight or OGTT data (n = 63). After excluding women who received any forms of GDM treatment during pregnancy (n = 578), a sample of 10,486 pregnant women with GDM was included in the analysis. The flowchart is shown in Supplementary Fig. 1. Overall, the average maternal age was 31.2 ± 4.6 years, and more than 40% of the women were unemployed. More than two-thirds of women had a healthy BMI, and nearly 20% were overweight or obese. A total of 35.5% of the women had a gravidity ≥ 3, and nearly 50% were multiparous. For gestational weight gain, more than half of the women gained more than the recommended weight gain: 37.2% had a weight gain above and 13.2% had a weight gain below the recommended weight gain. Maternal age, maternal height, prepregnancy weight, prepregnancy BMI, and oral glucose tolerance plasma glucose levels decreased with increasing BMI quartiles, while maternal weight at delivery, total gestational weight gain, gestational age, and birth weight showed the opposite trend(*P* < 0.001)(Table 1).

**Table 1:** Basic characteristics of the study population by maternal body mass index(BMI) change Quartile

Variable	Overall	BMI change Quartile				*P* value
	Total(n = 10,486)	Q1(n = 2622)	Q2(n = 2620)	Q3(n = 2622)	Q4(n = 2622)	
**Maternal age(years)**	31.2 ± 4.6	32.3 ± 4.6	31.6 ± 4.6	31.0 ± 4.6	29.9 ± 4.5	< 0.001
**Employment**						< 0.001
Employed	6177 (58.9)	1621 (61.8)	1625 (62)	1528 (58.3)	1403 (53.5)	
Unemployed	4309 (41.1)	1001 (38.2)	995 (38)	1094 (41.7)	1219 (46.5)	
**Education level**						< 0.001
Unversitiy and above,(%)	2524 (24.1)	776 (29.6)	683 (26.1)	623 (23.8)	442 (16.9)	
Blow unverisity,(%)	1846 (70.4)	1937 (73.9)	1999 (76.2)	2180 (83.1)	1846 (70.4)	
**Maternal Height(cm)**	159.7 ± 5.0	160.0 ± 5.3	159.6 ± 4.8	159.6 ± 4.9	159.3 ± 4.8	< 0.001
**Pre-pregnancy Weight(kg)**	55.3 ± 8.5	59.2 ± 9.5	55.0 ± 8.0	53.9 ± 7.8	53.0 ± 7.1	< 0.001
**Pre-pregnancy BMI(kg/m2)**	21.7 ± 3.1	23.1 ± 3.5	21.6 ± 2.9	21.1 ± 2.8	20.9 ± 2.6	< 0.001
Underweight(BMI < 18.5)(%)	1399 (13.3)	184 (7)	352 (13.4)	412 (15.7)	451 (17.2)	
Normal weight(BMI 18.5 ~ 23.9)(%)	7021 (67.0)	1500 (57.2)	1790 (68.3)	1853 (70.7)	1878 (71.6)	
Overweight or Obesity(BMI ≥ 24.0)(%)	2066 (19.7)	938 (35.8)	478 (18.2)	357 (13.6)	293 (11.2)	
**Oral glucose tolerance test**						
Fasting plasma glucose level(mmol/L)	5.6 ± 2.8	5.9 ± 3.7	5.5 ± 2.6	5.4 ± 2.5	5.4 ± 2.2	< 0.001
1 h plasma glucose level(mmol/L)	10.3 ± 2.3	10.5 ± 2.2	10.3 ± 2.3	10.2 ± 2.2	10.2 ± 2.4	< 0.001
2 h plasma glucose level(mmol/L)	8.6 ± 2.0	8.9 ± 2.0	8.6 ± 2.0	8.5 ± 1.9	8.3 ± 2.1	< 0.001
**Gravidity**						< 0.001
1	3644 (34.8)	740 (28.2)	849 (32.4)	952 (36.3)	1103 (42.1)	
2	3122 (29.8)	855 (32.6)	815 (31.1)	743 (28.3)	709 (27)	
≥ 3	3720 (35.5)	1027 (39.2)	956 (36.5)	927 (35.4)	810 (30.9)	
**Parity**						< 0.001
Primiparity,(%)	5409 (51.6)	1134 (43.2)	1282 (48.9)	1363 (52)	1630 (62.2)	
Multiparity,(%)	5077 (48.4)	1488 (56.8)	1338 (51.1)	1259 (48)	992 (37.8)	
**Delivery**						
Maternal Weight at delivery(kg)	67.9 ± 8.6	66.4 ± 9.2	66.3 ± 8.2	67.8 ± 8.2	71.1 ± 8.0	< 0.001
Total gestational weight gain(kg)	12.6 ± 4.4	7.2 ± 2.3	11.2 ± 1.0	13.9 ± 1.2	18.1 ± 2.7	< 0.001
Gestational age (weeks)	38.64 ± 1.50	38.30 ± 1.84	38.60 ± 1.44	38.74 ± 1.31	38.92 ± 1.29	< 0.001
Birth weight (g)	3299.7 ± 464.1	3184.4 ± 499.1	3286.6 ± 449.9	3336.7 ± 434.5	3391.1 ± 445.1	< 0.001
**Gestational weight gain category (%)**						< 0.001
As recommended	5197 (49.6)	1320 (50.3)	2254 (86)	1512 (57.7)	111 (4.2)	
Below recommended	1385 (13.2)	1285 (49)	99 (3.8)	1 (0)	0 (0)	
Above recommend	3904 (37.2)	17 (0.6)	267 (10.2)	1109 (42.3)	2511 (95.8)	

Date are presented as mean ± standard deviation for continuous variables and N(%) for categorical variables

BMI:body mass index

### Effects of BMI changes on LGA

**Fig. 1:**
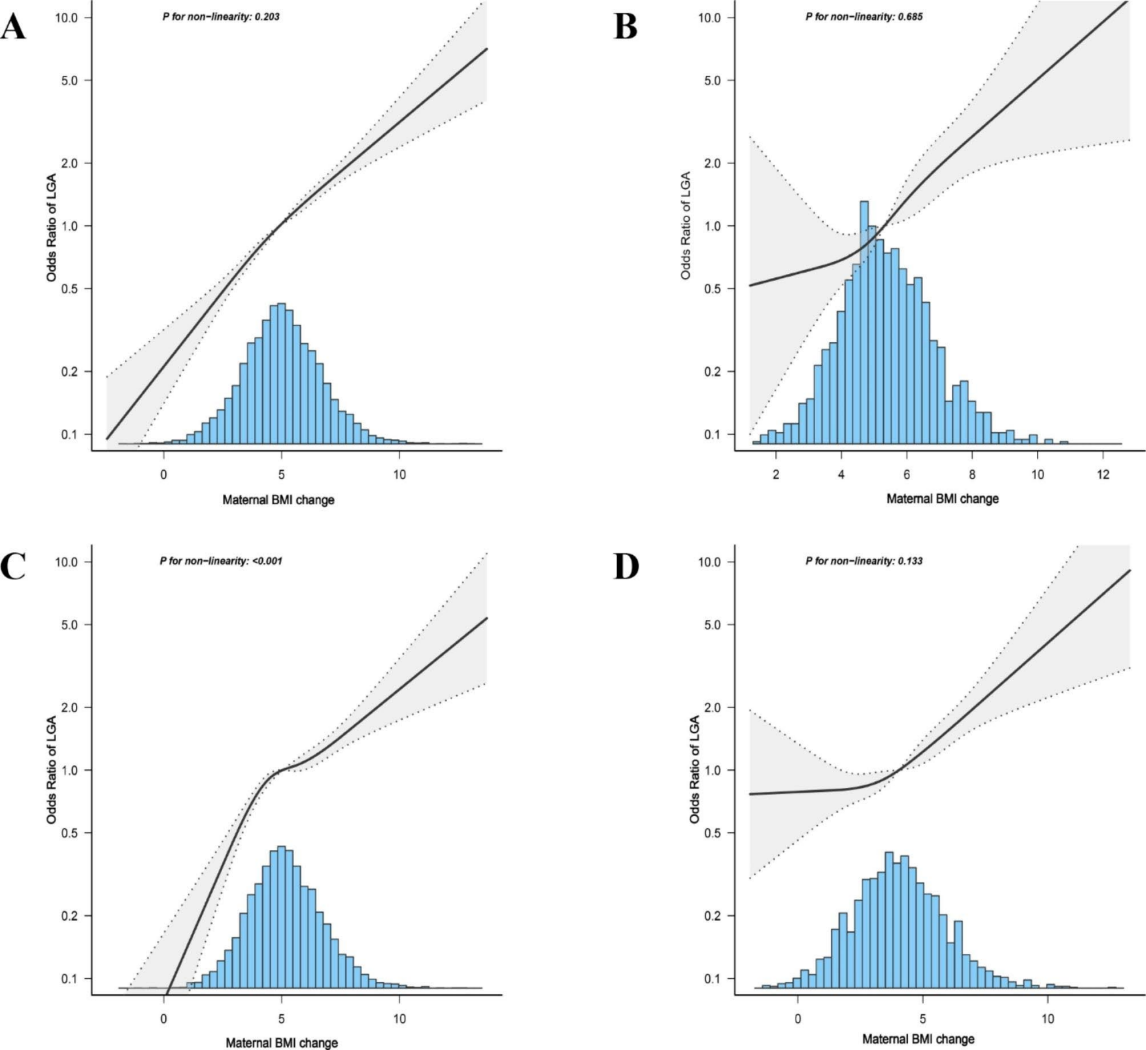
Dose-response relationship between maternal body mass index(BMI) change and large for gestational age(LGA). The solid line and shadow part represents the unadjusted probability and adjusted 95% confidence intervals. Adjusted for maternal age, education level, employment, gravidity, parity, gestational age at delivery, infant sex, oral glucose tolerance test level, pre-pregnancy body mass index, maternal glycemic control during the third trimester and serum triglyceride levels during the third trimester(B-D not adjusted for pre-pregnancy body mass index). (**A**) All women; (**B**) Underweight; (**C**) Normal weight; (**D**) Overweight and obesity

The dose‒response relationship between BMI changes and the probability of LGA is shown in Fig. 1A. An increasing trend of LGA with increasing BMI with or without adjusted confounding factors was observed(*P* for nonlinearity = 0.203).After classifying the prepregnancy BMI, we also found noticeable affiliated trends of BMI changes and the probability of LGA with or without adjusted confounding factors, and the *P* for nonlinearity was > 0.05 except for the normal weight category(*P* < 0.001)(Fig. 1B-D).

Table 2 shows the association of BMI changes with LGA determined by logistic regression analysis. After adjusting for maternal age, education level, employment, gravidity, parity, gestational age at delivery, and infant sex(Model 1), a 1-unit and a 1-SD increase in BMI increased the risk of LGA by 1.18 and 1.33 times, respectively. Similarly, the risk of LGA increased as the BMI change quartile increased, and the highest risk was observed in the last group (OR: 1.96; 95% CI: 1.69 ~ 2.27) compared with the first group (*P* < 0.001). Moreover, after adjusting for additional confounding factors (oral glucose tolerance test level, prepregnancy body mass index, maternal glycaemic control, andmaternal triglycerides during the third trimester), these findings remained, and the ORs increased (*P* for trend < 0.001).

**Table 2:** Association of maternal body mass index(BMI) change with large for gestational age(LGA).

Variable	LAG(%)	Crude OR(95%CI)	P value	Model1^a^			Model2^b^			Model3^c^	
				Adjusted OR(95%CI)	P value	Adjusted OR(95%CI)		P value	Adjusted OR(95%CI)		P value
BMI change, per unit	2111(20.1)	1.16(1.13 ~ 1.20)	< 0.001	1.18 (1.15 ~ 1.22)	< 0.001	1.19(1.15 ~ 1.22)		< 0.001	1.27 (1.23 ~ 1.31)		< 0.001
BMI change, per SD	2111(20.1)	1.329(1.23 ~ 1.36)	< 0.001	1.33 (1.26 ~ 1.4)	< 0.001	1.34(1.27 ~ 1.41)		< 0.001	1.51 (1.43 ~ 1.59)		< 0.001
BMI change, Quintile1	391 (14.9)	1(Ref)		1(Ref)		1(Ref)			1(Ref)		
BMI change, Quintile2	501 (19.1)	1.35 (1.17 ~ 1.56)	< 0.001	1.35 (1.17 ~ 1.57)	< 0.001	1.38(1.19 ~ 1.60)		< 0.001	1.75 (1.5 ~ 2.04)		< 0.001
BMI change, Quintile3	571 (21.8)	1.59 (1.38 ~ 1.83)	< 0.001	1.6 (1.39 ~ 1.86)	< 0.001	1.65(1.43 ~ 1.91)		< 0.001	2.23 (1.91 ~ 2.60)		< 0.001
BMI change, Quintile4	648 (24.7)	1.87 (1.63 ~ 2.15)	< 0.001	1.96 (1.69 ~ 2.27)	< 0.001	2.01(1.73 ~ 2.32)		< 0.001	2.84 (2.43 ~ 3.32)		< 0.001
Trend.test	2111(20.1)	1.22 (1.17 ~ 1.28)	< 0.001	1.24 (1.19 ~ 1.30)	< 0.001	1.25(1.19 ~ 1.31)		< 0.001	1.39 (1.32 ~ 1.45)		< 0.001

^a^Model 1 adjusted for maternal age, education level, employment, gravidity, parity, gestational age at delivery, and infant sex;

^b^Model 2 adjusted for maternal age, education level, employment, gravidity, parity, gestational age at delivery,infant sex, and Oral glucose tolerance test level;

^c^Model 3 adjusted for maternal age, education level, employment, gravidity, parity, gestational age at delivery,infant sex, Oral glucose tolerance test level, pre-pregnancy body mass index, maternal glycemic control and maternal triglyceride during the third trimester

### Stratification analysis on the association of BMI changes with LGA

**Fig. 2:**
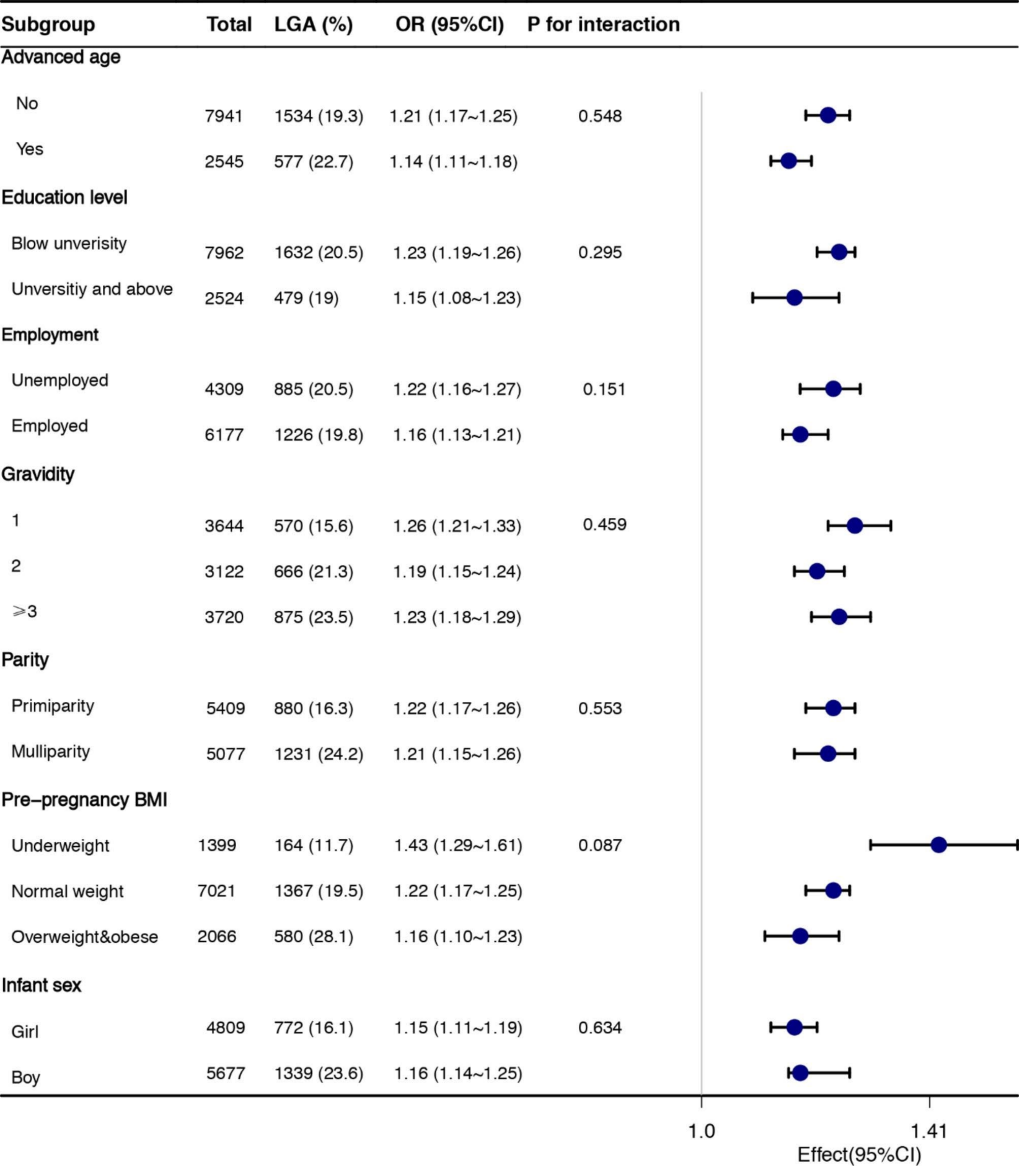
Stratification analysis on the association between maternal body mass index (BMI) change and large for gestational age (LGA).

Further analysis of the association of BMI changes with LGA stratified by advanced maternal age (yes, no), educational level (below university, university and above), employment(employed, unemployed), gravidity(1,2, ≥ 3), parity(primiparous, multiparous), prepregnancy BMI(underweight, normal weight, overweight and obesity), and infant sex(girl, boy) was performed. BMI changes (1-unit increase) were positively associated with the risk of LGAin all subgroups, and no significant interaction was found between the subgroups and therisk of LGA (Fig. 2).

### ROC curve analyses of BMI changes predicting LGA

**Fig. 3:**
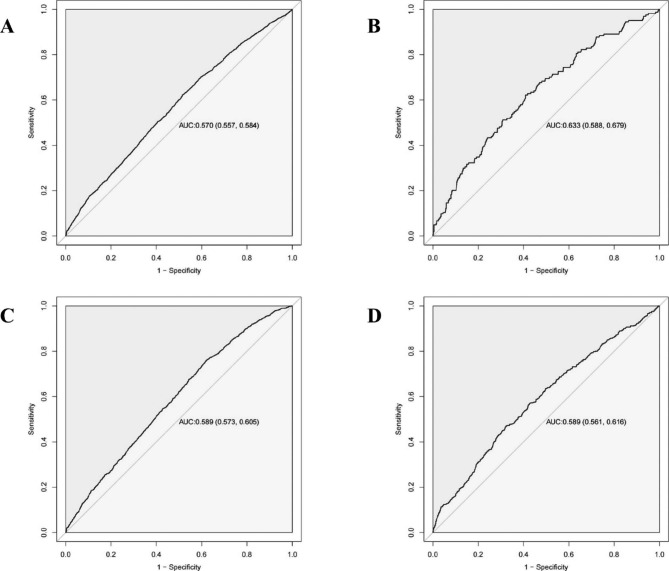
Receiver operating characteristic (ROC) curve analyses of maternal body mass index (BMI) change predicting large for gestational age (LGA). (**A**) All women; (**B**) Underweight; (**C**) Normal weight; (**D**) Overweight and obesity

Receiver operating characteristic (ROC) curve analyses of BMI changes were performed to predict LGA. In analyses ofthe entire population and further classified by prepregnancy BMI categories, BMI changesshowed significant areas under the ROC curve (AUCs, all *P* < 0.05), and the AUC of the BMI change for LGA was the 0.570 (95% CI: 0.557 ~ 0.584) in the entire study population. The best optimal predictive cut-off was 4.922, with a sensitivity of 0.622 and specificity of 0.486. For BMI categories, the AUCs (95% CI) for the underweight, normal weight, and overweight and obesity groups were 0.633(0.588 ~ 0.679), 0.589(0.573 ~ 0.605), and 0.589(0.561 ~ 0.616), respectively, and the best optimal predictive cut-offs were 5.313, 5.047, and 4.049, respectively, as shown in Fig. 3; Table 3.

**Table 3:** ROC analysis of the BMI change for predicting LGA

BMI category	AUC(95%CI)	Threshold	Sensitivity	Specificity	Accuracy
All women	0.570(0.557 ~ 0.584)	4.922	0.622	0.486	0.513
Underweight	0.633(0.588,0.679)	5.313	0.622	0.591	0.595
Normal weight	0.589(0.573 ~ 0.605)	5.047	0.764	0.376	0.451
Overweight and obesity	0.589(0.561 ~ 0.616)	4.049	0.471	0.676	0.619

AUC: area under curve

## Discussion

In the current study, we evaluated the associations between maternal BMI changes during pregnancy and LGA among Chinese women with GDM. We demonstrated that maternal BMI changes could be a useful predictor of the incidence of LGA in singleton pregnant women with GDM, and the AUC (95% CI) for LGA in all study populations was 0.570(0.557 ~ 0.584). The best optimal predictive cut-off was 4.922, with a sensitivity of 0.622 and specificity of 0.486. Further classified by prepregnancy BMI, maternal BMI changeswerestill significantly linked with the risk of incident LGA, and the best optimal predictive cut-off value decreased from the underweight group to the overweight and obesity group.

Normalizing foetal growth is important in the management of GDM, and excessive gestational weight gain leads toan increased risk for LGA in newborns. The association between excessive gestational weight gain and foetal LGA in women with GDM has been reported in several studies. Ronit Koren et al. evaluated 673 women with GDM and revealed that LGA newborns were significantly more prevalent in women with excessive gestational weight gain than in patients with appropriate and insufficient gestational weight gain [8]. Another multicentric retrospective study of 18,961 pregnant women with GDM demonstrated that excessive gestational weight gain was associated with an increased risk of LGA in infants regardless of prepregnancy BMI [27]. Maternal weight gain, as a risk factor, has been revealed to be associated with foetal overgrowth. A systematic review published in 2009 confirmed the associations between excessive gestational weight gain and increased birth weight and foetal growth [28]. However, researchers are still unable to determine an appropriate weight gain for women with GDM. Additionally, it should be noted that the target of the gestational weight gain guidelines focus on balancing the risk of adverse pregnancy outcomes. Foetal growth directly or indirectly reflects most other perinatal outcome components; thus, the amount of gestational weight gainedthat reducesthe incidence of LGA represents the best optimal weight gain, to some extent. Additionally, the length of pregnancy should be considered in determining the target weight gain. In this study, we suggested the maternal BMI change as a new measurement in the context of maternal height to monitor weight gain during pregnancy, which is important for an individualized approach for each pregnant woman, and health care providers can estimate the recommended BMI change and recalculate the appropriate weight gain in kilograms according to maternal height. The current study revealed that the risk of LGA increased with increasing maternal BMI in pregnant Chinese women with GDM in all prepregnancy BMI categories. Furthermore, the analysis stratified by advanced maternal age, educational level, employment, gravidity, parity, prepregnancy BMI, infant sex did not affect our results. Maternal BMI changes are feasible for stratifying the risks of pregnancy outcomes in many studies. A study focused on 1205 women who were underweight before becoming pregnant showed that a BMI change of 4–5 kg/m^2^was associated with a lower prevalence of SGA newborns [29]. Morikawa et al. [21] determined that a BMI change of more than 6.0 kg/m^2^ was an independent risk factor for delivering an infant with macrosomia. A multicentre retrospective study using electronic medical record data from Japan indicated that an annual increase in BMI was negatively associated with spontaneous preterm birth, and the spontaneous preterm birth recurrence rate was significantly lower in patients with an annual BMI change of ≥ 0.25 kg/m^2^ than in those with an annual BMI change of < 0.25 kg/m^2^ [18]. In this study, we present for the first time the idea of BMI changes in the context of female height as a new output measure. For a woman with normal weight and a height of 160 cm, the recommended weight gain range is 8.0–14.0 kg, and the recalculated appropriate weight gain in kilograms according to the BMI change threshold is less than 12.9 kg, whereas for a woman with a height of 180 cm, the recalculated weight gain is less than 16.4 kg. This means that according to the existing weight gain guidelines, for women with the same prepregnancy BMI, an identical optimal gestational weight gain is recommended for a woman with a height of 160 cm and women with a height of 180 cm. Therefore, our findings may be an important step forward in personalizing gestational weight gain, which is especially important for very tall and very short women. This also means that BMI changes can be combined with Chinese guidelines to improve the approach used in the clinic.

We also observed a decreasing maternal BMI change threshold for predicting LGA with incremental increases in prepregnancy BMI. Consistent with the present report, previous studies have reported a positive association between prepregnancy BMI and the risk of LGA in women with or without GDM [30–33].

There are several limitations to the present study. First, due to the single-centre retrospective design, our results are limited in generalizability and do not provide causal evidence for reducing the risk of LGA in women with GDM and call for a multicentre study on other populations. Second, the exclusion of women who were treated for serious hyperglycaemia or heavier prepregnancy weight may affect the results of the study. Third, due to the retrospective design, some confounding factors, such as family history and lifestyle, were not available, which may underestimate the incidence of LGA. Despite these limitations, our study is beneficial for the prevention of foetal overgrowth in pregnant women with GDM. It provides new ideas for efforts to find a method to calculate the optimal gestational weight gain for each pregnant woman. Maternal overweight and obesity, GDM, and excessive gestational weight gain are all independent risk factors forfoetal overgrowth, including LGAand macrosomia, which can lead to diabetes, obesity, metabolic syndrome, and asthma in adulthood [3]. Preventing pregnancy weight gain may be more feasible than preventing obesity and GDM. Studies have indicated that the most effective interventions to prevent pregnancy weight gain are closely related to lifestyle. These interventions mainly include daily diet control, frequent weight measurement, behavioural adjustment, and ongoing contact with health care providers [34]. Furthermore, preventing excessive gestational weight gain will also avoid excessive postpartum weight retention, which can in turn impact subsequentpregnancies.

## Conclusions

In conclusion, maternal BMI changes are closely related to the risk of LGA in women with GDM. Maternal BMI changes,in conjunction with the Chinese guidelines, may reduce the incidence of LGA in patients with GDM in theclinic. Future multicentre prospective population studies are needed for further verification.

## Electronic supplementary material

Below is the link to the electronic supplementary material.

Supplementary Fig. 1: Flowchart of Subject selection

## Data Availability

The datasets used and/or analysed during the current study are available from the corresponding author on reasonable request.

## Abbreviations

GDM: Gestational diabetes mellitus
LGA: Large for gestational age
BMI: Body mass index
OGTT: Oral glucose tolerance test
OR: Odd ratios
CI: Confidence intervals
SD: Standard deviation
ROC: Receiving-operating characteristic
AUC: Areas under the curve
IOM: Institute of Medicine
