# Supplementary figures and images for: Maternal body mass index and risk of fetal overgrowth in women with gestational diabetes Mellitus in Southeast China: a retrospective cohort study
Source: Diabetol Metab Syndr. 2023 Jun 8;15:121. doi: 10.1186/s13098-023-01093-y (PMC10249232; doi:10.1186/s13098-023-01093-y)

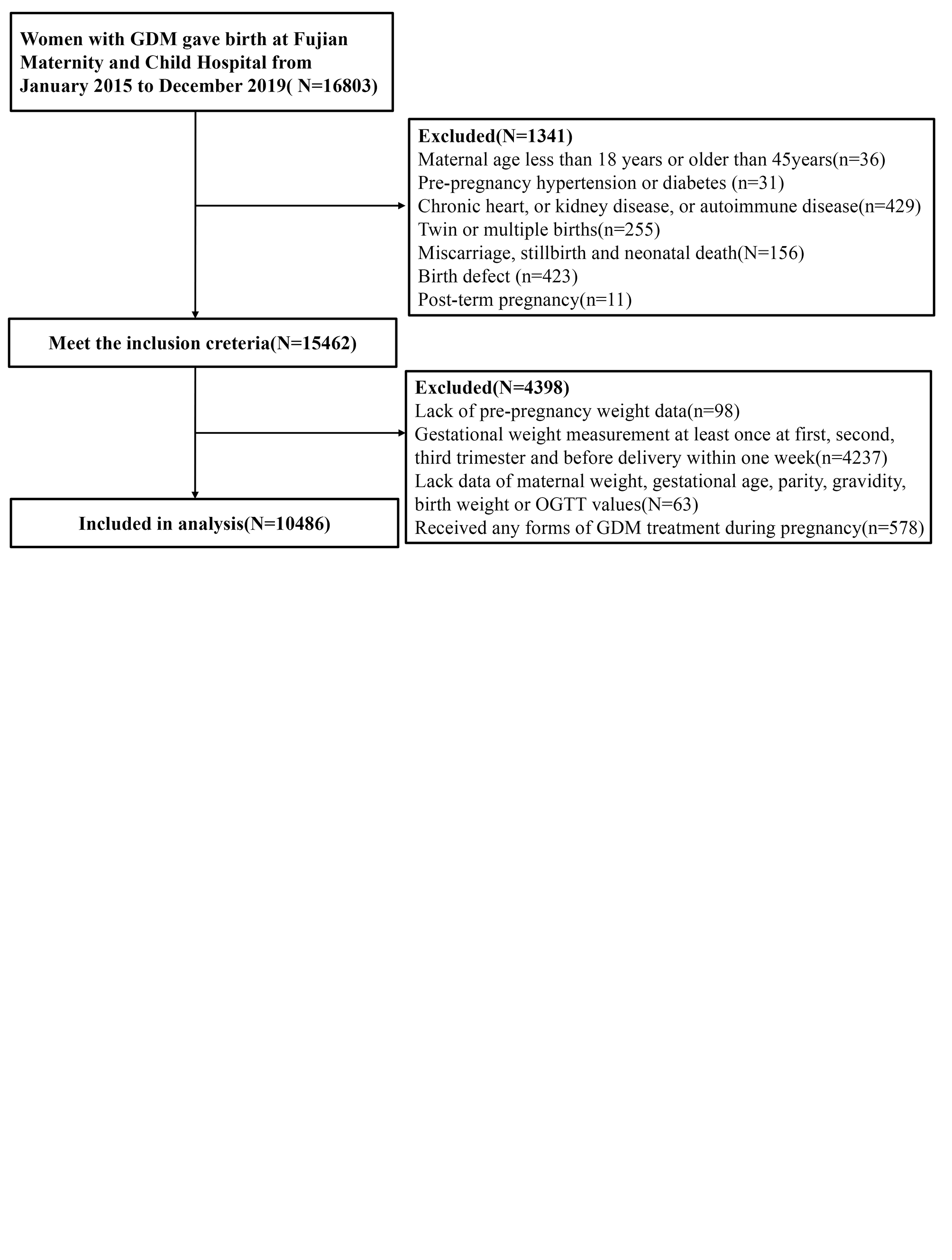

Supplement: Supplementary file 1 — Supplementary Fig. 1: Flowchart of Subject selection [file 13098_2023_1093_MOESM1_ESM.tif]
